# Patients’ costs, socio-economic and health system aspects associated with malaria in pregnancy in an endemic area of Colombia

**DOI:** 10.1371/journal.pntd.0006431

**Published:** 2018-05-02

**Authors:** Elisa Sicuri, Azucena Bardají, Sergi Sanz, Sergi Alonso, Silke Fernandes, Kara Hanson, Myriam Arevalo-Herrera, Clara Menéndez

**Affiliations:** 1 ISGlobal, Hospital Clínic—Universitat de Barcelona, Barcelona, Spain; 2 Health Economics Group, Department of Infectious Disease Epidemiology, School of Public Health, Imperial College London, London, United Kingdom; 3 CIBER Epidemiología y Salud Pública (CIBERESP), Madrid, Spain; 4 Biostatistics Unit, Department of Public Health, Faculty of Medicine, University of Barcelona, Barcelona, Spain; 5 Centro de Investigação em Saúde de Manhiça (CISM), Maputo, Mozambique; 6 Department of Global Health and Development, London School of Hygiene and Tropical Medicine, London, England; 7 Caucaseco Scientific Research Center/Universidad del Valle, Cali, Colombia; Federal University of Agriculture, NIGERIA

## Abstract

Malaria in pregnancy threatens birth outcomes and the health of women and their newborns. This is also the case in low transmission areas, such as Colombia, where *Plasmodium vivax* is the dominant parasite species. Within the Colombian health system, which underwent major reforms in the 90s, malaria treatment is provided free of charge to patients. However, patients still incur costs, such as transportation and value of time lost due to the disease. We estimated such costs among 40 pregnant women with clinical malaria (30% *Plasmodium falciparum*, 70% *Plasmodium vivax*) in the municipality of Tierralta, Northern Colombia. In a cross-sectional study, women were interviewed after an outpatient or inpatient laboratory confirmed malaria episode. Women were asked to report all types of cost incurred before (including prevention), during and immediately after the contact with the health facility. Median total cost was over 16US$ for an outpatient visit, rising to nearly 30US$ if other treatments were sought before reaching the health facility. Median total inpatient cost was 26US$ or 54US$ depending on whether costs incurred prior to admission were excluded or included. For both outpatients and inpatients, direct costs were largely due to transportation and indirect costs constituted the largest share of total costs. Estimated costs are likely to represent only one of the constraints that women face when seeking treatment in an area characterized, at the time of the study, by armed conflict, displacement, and high vulnerability of indigenous women, the group at highest risk of malaria. Importantly, the Colombian peace process, which culminated with the cease-fire in August 2016, may have a positive impact on achieving universal access to healthcare in conflict areas. The current study can inform malaria elimination initiatives in Colombia.

## Author summary

Malaria in pregnancy is a harsh and undesirable illness and is the cause of adverse effects on birth outcomes and on the health of women and newborns. Despite the low transmission, the predominance of *Plasmodium vivax* over *Plasmodium falciparum* and free treatment, estimated costs incurred by pregnant women seeking malaria care in an endemic area of Northern Colombia are considerable. Importantly, these costs are likely to represent only one of the constraints that women face when seeking treatment in an area characterized, at the time of the study, by armed conflict, displacement, and high vulnerability of indigenous women, the group at highest risk of malaria in the area. Important advances may result from the current peace process, potentially able to support the efforts made since the 90s to reform the health system towards achieving universal health coverage. Within this context, the current study can inform malaria elimination initiatives in Colombia.

## Introduction

Malaria is a harsh, undesirable and life-threatening disease and, if experienced during pregnancy, can cause adverse effects on birth outcomes and on the health of women and newborns [1]. Such effects encompass long term disabilities and, at the aggregate level, are likely to slow down considerably the economic development of endemic areas [2–4]. In the short term, a malaria in pregnancy (MiP) episode may produce a considerable shock to households’ budgets [5].

In low endemicity areas, including Colombia where *P*. *vivax* predominates, little information is available on the epidemiology and the socio-economic aspects of malaria. In Colombia, few studies have examined the socio-economic determinants and impacts of the infection [6–9]. All the published studies depict malaria in Colombia as the disease of the poorest people living in rural areas. However, only one study offered estimates of malaria costs to families [6], while to date, no published study has assessed the socio-economic burden of MiP in the country.

The socio-economic aspects of disease are influenced by the characteristics of the health system. This is particularly true for Colombia where the health system has been under continuous reform for the past 25 years. Major reforms were undertaken in 1993 with the aim of increasing health system efficiency and access to healthcare, which was very skewed towards formal workers and left informal workers, a large proportion of the active population, unprotected [10]. Under a managed competition model, the reform created two schemes, contributory and subsidised: the former targeting formal employees and people able to pay, financed by mandatory contribution; the latter targeting people with no ability to pay, funded by the contributory scheme and by other sources such as general taxation. Eligibility for the subsidised regimen is determined through a socio-economic index called SISBEN (*Sistema de Identificación de Beneficiarios; Beneficiaries Identification System*) that classifies the population into six strata based on several socio-economic indicators [11]. The lowest two strata (the poorest) are eligible for the subsidised scheme and patients pay either nothing or a small fee for the healthcare they receive.

Although the 1993 reform effectively increased health insurance coverage, there were discordant views on the existence of pockets of inequity the reform may have left: according to some researchers the increase in insurance coverage did not completely translate into healthcare coverage [12–14]. Recently, under the principle of the universal right to health, citizens in search of (expensive) health interventions denied by the private insurers have brought legal action (*tutelas*) to the Constitutional Court. Denied health services were finally funded by the Governmental Solidarity and Guarantee Fund (*Fosyga*) leading to sharp increases in public expenditure, to the extent that a recent government decree declared a “social emergency” due to a health financing crisis [15, 16].

Some studies have assessed the regressivity or progressivity of the Colombian health financing system but still little information is available on the out-of-pocket expenditures incurred, particularly in non-urban areas, among communities whose main activity is agriculture or livestock [17].

In addition, armed conflict in certain rural areas has led to additional challenges for healthcare provision with women and ethnic minorities being at highest risk [18, 19]. To address this, a series of public health interventions have targeted women of ethnic minorities living in rural conflict areas which are often also malaria endemic areas [20]. Living in rural and conflict-affected areas has been found to be a risk factor for catastrophic expenditures in Colombia [21].

According to the National Public Health Plan, malaria in Colombia is a “disease of public health interest” and all the interventions, including both prevention and treatment, are free of charge to the population, irrespective of health scheme affiliation [22]. However, despite zero medical costs, patients still incur non-medical expenses, such as transportation, and indirect costs, such as the value of time lost because of the illness.

The aim of this study was to estimate the costs associated with MiP in a rural area of the Cordoba department, Northern Colombia, the area with the highest malaria burden in the country [23–25]. At the time of the study, the area was characterized by armed conflict and high vulnerability of ethnic minorities.

## Methods

### Ethics statement

This study was conducted in the context of the PregVax study. This was a health-facility based cohort study aimed to estimate the burden of *P*. *vivax* infection in pregnancy in five endemic countries, including Colombia. The ethics statement for PregVax is published elsewhere [26]. In addition, the economic study (ECO_PregVax) was approved by the Ethics Committee of the Hospital Clinic of Barcelona, Spain.

All adult subjects provided written informed consent prior to participating in ECO_PregVax and, if younger than eighteen years of age, a parent or guardian provided written informed consent on the child’s behalf.

### Study area

This study was carried out in the municipality of Tierralta, department of Cordoba, a malaria endemic area of the Colombian territory [23].

The municipality has an area of nearly 5,000 km^2^ with about 90,000 inhabitants, 44.4% of whom live in rural areas, 55.6% in urban or semi-urban areas, and about 2% of the population is indigenous. The indigenous population Embera Katío used to live in a protected area located on the high basin of the Sinú river, administratively pertaining partly to the municipality of Tierralta and partly to Ituango (Antioquia department). Due to both the construction of a hydroelectric power plant that came into operation in the year 2000 and to the armed conflict, the Embera Katío were forced to spread all over the municipality.

The main economic activities within the municipality are cattle farming, agriculture, logging and fishing. The Gross Domestic Product per capita of Cordoba department for the year 2014 was about 3,900 US$ [27].

According to the national public health surveillance system, in 2010, out of a total of 117,108 malaria cases reported in Colombia, more than 20,000 (17.2%) were registered in Córdoba and most of them (over 14,000) were due to *P*. *vivax*. Of the cases reported in Cordoba, 29 were in pregnant women [23]. In a village of the municipality of Tierralta, the prevalence of *Plasmodium spp*. infection was 17.9% (38/212; 95% CI: 12.5–23.3%) [24]. The annual parasite index (API) recorded between 2008 and 2012, was of 44.0 cases/1,000 habitants. Based on this, Tierralta was considered as a high risk malaria area in comparison with two other endemic areas of the country, namely Buenaventura (Valle del Cauca) and Tumaco (Nariño), with API of 6.0 cases/1,000 habitants and 7.7 cases/1,000 habitants, respectively [25].

In Tierralta malaria and respiratory diseases are perceived as the most common health problems in the population. The lack of environmental interventions is recognized as the major cause of malaria [28]. In a knowledge, attitudes and practices study undertaken in three regions (Tierralta, Buenaventura and Tumaco) it was observed that most of the population uses insecticide-treated nets (ITNs) to protect themselves from malaria, but over 75% of the people in Tierralta do not use any tool or strategy to prevent malaria transmission outdoors [25].

Data were collected in the catchment area of San José hospital, a public primary level health facility offering a range of services, including outpatients, immunization, antenatal care, laboratory, pharmacy, dentistry, radiology services, emergencies and inpatients (delivery, pediatric and adults). San José hospital directly administers 16 aid posts spread across the municipality where a medical doctor attends patients for a few hours every week. Aid posts help increase access to healthcare considering that traveling to the hospital from the villages takes up to 8 hours by combined transportation (usually motorbike plus boat) and the journey may include crossing the Sinú river.

### Study design

The PregVax study enrolled 2,043 pregnant women at the antenatal clinic of the San José hospital between April 2009 and June 2011 and followed them up until delivery. Prevalence of MiP by microscopy was 1.2% detected either at ANC follow up visits or at out- or in-patient wards during the study period [26].

Between July and August 2011 a subset of women enrolled in the main study and diagnosed with any *Plasmodium* species infection either during pregnancy or during post-partum, were invited to participate in this economic study. As many women as was feasible, given the conditions of insecurity in the area, were reached and interviewed at their homes. After written informed consent was given, a questionnaire was administered by a trained fieldworker. We collected personal information, time lost because of the illness, data on use and costs for the treatment and prevention (bed net, skin repellent, insecticide) associated with MiP, including direct (medical and non-medical) costs incurred at the health facility. Women were asked about any previous treatments sought for the symptoms associated with the same malaria episode. Characteristics of women included in this study were compared with those of women enrolled in the epidemiological study but not enrolled in the economic study (both malaria infected and non-infected).

### Data analysis

The data analysis and presentation of results were similar to a previous study carried out in Brazil [5]: direct costs (financial costs) were broken down into medical, transportation and other costs (food, phone calls, etc.). Indirect costs were calculated by multiplying reported time lost by the legal minimum wage in force in Colombia in the year 2012 (Colombian pesos $566,700, monthly, corresponding to US$ 307) [29]. The exchange rate was 1,848.14 Colombian pesos to the dollar [30]. Patient costs incurred at the San Jose hospital, as well as for any treatment sought earlier for the same episode, were calculated.

Due to the skewness of the distribution, the median and the interquartile range (IQR) were used to report cost estimates. Mean values are also shown to provide a comprehensive description of cost distributions. Bootstrap simulation with 1000 replications was carried out to resolve distribution skewness [31, 32].

The place of residence of each study woman (neighbourhood) was located on digital maps and distance to the health facility was calculated linearly, due to the impossibility of tracing the itinerary women may have followed to obtain treatment.

For this study, women were approached only once. However, some women experienced more than one malaria episode during the follow-up period. To produce an estimate of the total cost of MiP, the database generated was merged with the data from the main epidemiological study. For the women with more than one episode, the cost estimated for one episode was multiplied by *N* episodes, assuming that cost per episode was constant.

Data were analyzed with STATA version 12.0 (Stata/SE 12.0 Stata corporation, College Station, TX, USA).

## Results

A total of 69 women enrolled in the main epidemiological study had a malaria infection identified either by microscopy or by PCR during pregnancy and/or at delivery. Of these, 27 were included in this economic study. Additionally, 13 women from the main study who had malaria post-partum were also enrolled for a total of 40 women. There were no statistically significant differences in terms of age and ethnic group between the 40 women included in this economic study and the pregnant women with malaria from the main study who were not involved in this evaluation (N = 29): (age: t = 0.0778, Pr (¦T¦ > ¦t¦ = 0.9382); ethnic group: Pearson chi2 (2) = 0.2119, Pr 0.899). Similarly, age and ethnic group differences were not significantly different between the 40 study women and those from the main study who were not included (N = 2003): (age: t = -1.3029, Pr (¦T¦ > ¦t¦ = 0.1928; ethnic group: Pearson chi2 (2) = 1.1488, Pr 0.886).

Table 1 shows that most of the 40 study women were aged between 20 and 29 years (47.5%) but many were below 20 years (35%) and a few were 30 years old or more (17.5%). The majority had already one to three children (52.5%). The great majority were common-law wives (92.5%). Most of the women lived in the rural areas (72.5%) and all of them declared their main activities to be household activities and child-care.

**Table 1:** Baseline characteristics of study subjects (N = 40).

	N	%
Age group		
15–19	14	35
20–29	19	47.5
30–39	7	17.5
Marital Status		
Single	2	5
Common-law wife	37	92.5
Married	1	2.5
Living área		
Semi-urban	11	27.5
Rural	29	72.5
Number of children (excluding the current one)		
0	15	37.5
1 to 3	21	52.5
4 to 5	4	10
Main activity		
Household activities/childcare	40	100
Health system regimen		
Subsidised level 1	33	82.5
Beneficiaries identification system—Sistema de identificación de beneficiarios (SISBEN)	6	15
Contributory	1	2.5
Health promotion entity—entidad promotora de salud (EPS)*		
CAPRECOM	4	12
EMDIS	21	62
MANEXKA	8	23
SALUCOOP	1	3

*This information is reported from the 34 women not subscribed to SISBEN regimen

Only one woman in this sample was enrolled in the contributory regimen while the remaining 39 were either part of the subsidised (1^st^ level) or not yet assigned to the subsidised scheme but already assessed not to have ability to pay.

Most of the study women were affiliated to EMDI Salud, one of the main private not-for profit organizations managing subsidized health care in the department. Interestingly, 8 women were affiliated to Manexka, a health promoting entity (entidad promotora de salud—EPS) for indigenous population, highlighting that these 8 women belonged to the Embera Katío ethnic group.

Seventy percent of malaria cases were due to *P*.*vivax* and the remaining to *P*. *falciparum* (Table 2) [33]. Most cases were outpatients (67.5%). Bed nets (either insecticide treated or not), the only type of malaria preventative measure used, were reported by 26 women. Most women reached the hospital by motor transportation (82.5%) and some women took more than one type of transportation (boat plus something else). For most women (82%) it took up to two hours to reach the health facility, but for some the trip was longer (2–5 hours) and for one woman the journey lasted 48 hours (by animal transportation).

**Table 2:** Characteristics of malaria episodes (N = 40).

	N	%
**Type of malaria episodes (*Plasmodium* species)**		
*P*. *vivax*	28	70
*P*. *falciparum*	12	30
**Type of contact with health facility**		
Outpatient	27	67.5
Inpatient	13	32.5
**Malaria prevention tools used**		
Bed net	26	65
No specific prevention tools	14	35
**Transportation to the health facility**^**a**^		
Animal transportation	1	2.6
Motor transportation	32	82
Combined transportation	6	15.4
**Time taken to reach health facility (Minutes)**		
15–120	33	82.5
121–300	6	15
>300 (precisely 2880 minutes = 48h)	1	2.5
	**Median**	**IQR**
Distance from health facility^b^ (Km) N = 33	14.28	17.33
Duration of clinical symptoms (days)	5	4.5
Number of nights admitted if inpatient (N = 13)	3	2

^a^ One observation was missing on type of transportation to the health facility, despite reporting transportation cost >0

^b^ maximum distance was 38 km.

**Fig 1:**
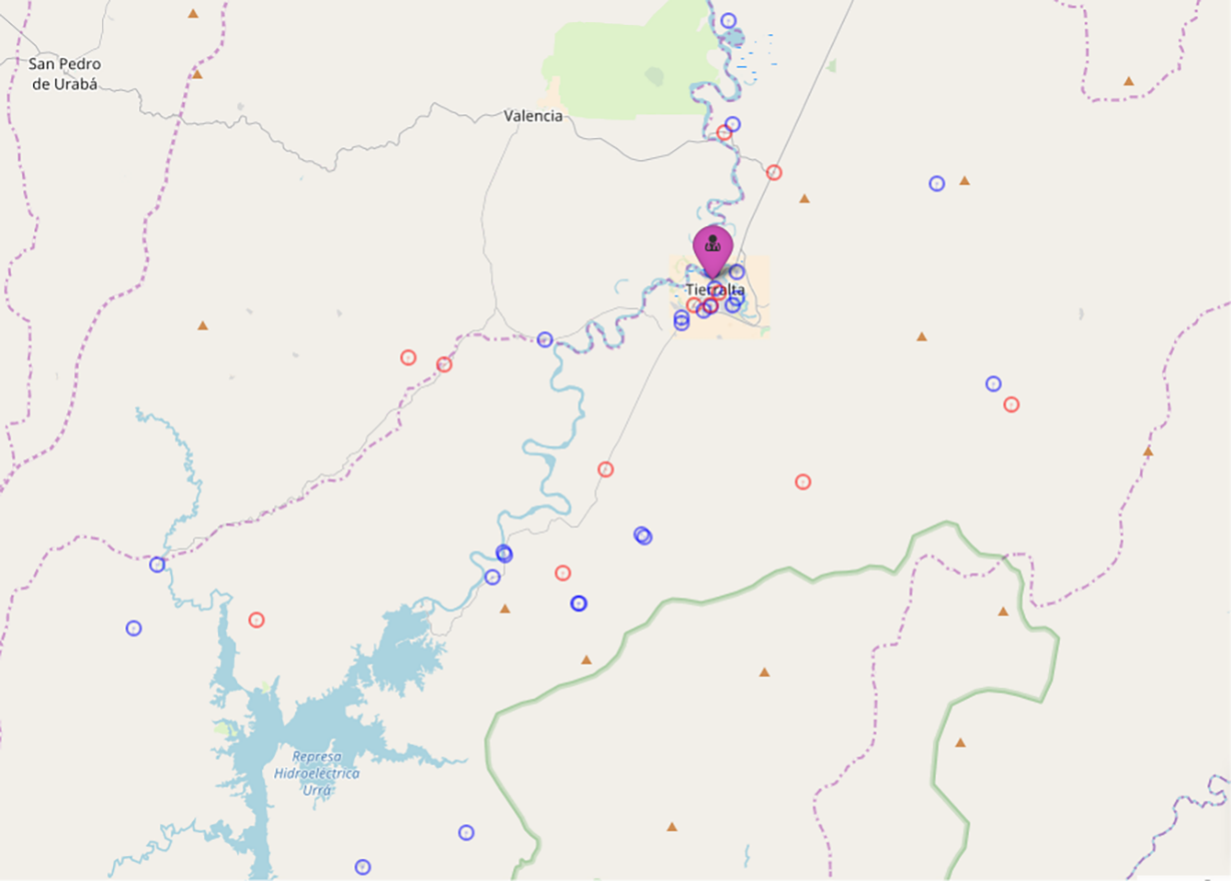
Residence of women included in the study. Red circles: *Plasmodium falciparum* malaria cases; Blue circles: *Plasmodium vivax* cases. Purple marker: San José hospital.

The place of residence of 39 women was represented on a map. One woman’s residence was not identified due to insufficient information. Median distance, estimated for 33 women, was of 14.28 km (IQR 17.33) (Fig 1). This is likely to be an underestimate as six women, despite being depicted on the map in areas far-away from the health facility (e.g. around Crucito), were not included in the distance estimate due to imprecise information: they were only able to provide the names of geographically spread neighbourhoods. Twelve women lived in town while the remaining were either far or very far from the health facility and some had to cross the river twice to reach a health consultation.

### Outpatient costs

Median prevention costs (all for bed nets), were US$ 0 (IQR 8.12). Only 14 women spent a positive amount to acquire a bed net (possibly because these were distributed for free either by the government or by non-governmental institutions) and 26 (65%) women declared they had slept under a bed net the night before the interview (Table 3). Only 25% of the interviewed women had sought previous treatment and the cost incurred was negligible (mean US$ 0.88, with a median and IQR of 0).

**Table 3:** Costs associated with malaria incurred by the pregnant women (US$ 2011).

	Outpatients N = 27			Inpatients N = 13		
	*P*. *vivax* = 24; *P*. *Falciparum* = 3			*P*. *vivax* = 24; *P*. *Falciparum* = 3		
	Mean	Median	IQR^a^	Mean	Median	IQR^a^
						
Prevention costs (N = 26 declared they slept under bed net last night)	3.75	0.00	8.12	2.53	0.00	0.00
Costs of previous symptomatic treatment sought (N = 10 sought previous treatment at a health centre)	0.40	0.00	0.00	1.87	0.00	0.00
Transportation costs to the health facility	16.07	12.99	23.80	12.82	10.82	17.32
Other costs at the health facility^b^	0.02	0.00	0.00	0.00	0.00	0.00
Medical costs for treatment at the health facility^c^	0.00	0.00	0.00	0.00	0.00	0.00
Indirect costs at this health facility^d^	9.13	7.29	3.64	10.55	9.11	3.64
Indirect costs whole episode (including previous symptomatic treatments)^e^	11.15	7.29	6.37	25.27	17.31	18.22
Total costs per episode at the health facility^f^	18.19	16.29	21.64	27.54	26.65	24.94
Total costs whole episode (including previous symptomatic treatments)^g^	29.75	28.93	20.39	54.68	54.33	49.09
*Bootstrap analysis (bootstrap based on 1000 replications)*						
Prevention costs	3.53	0.00	8.12	2.38	0	0
Total costs per episode at the health facility	18.00	16.28	21.64	27.66	26.65	24.94
Total costs whole episode (including previous symptomatic treatments)	29.42	28.93	20.39	56.23	54.33	54.31

a Interquartile range

b These include cost of food, drink and phone calls

c These include laboratory fees and drugs not provided at the health facilities

d These include the value of time lost at the health facility and money spent to contract a substitute in the main economic activity

e These include the value of time lost during the whole pattern of treatment sought and money spent to contract a substitute in the main economic activity

f These include all costs, direct and indirect, associated with treatment incurred at the health facility

g These include all costs, direct and indirect, associated with treatment incurred at the health facility plus direct and indirect costs previously incurred for the same sample

Transportation was a substantial source of cost, US$ 12.99 (IQR 23.80) and so were indirect costs of US$ 7.29 (IQR 10.55) at the health facility or US$ 7.29 (IQR 25.27) if the whole episode was considered. Estimation using a bootstrap with 1000 replications led to similar results.

### Inpatient costs

Cost of prevention and previous treatment had both median and IQR equal to 0; the mean values were US$ 2.53 and 1.87, respectively (Table 3). Transportation costs were US$ 10.82 (IQR 17.32). Indirect costs at the health facility were US$ 9.11 (IQR 3.64) or 17.31 (IQR 18.22) when the whole clinical episode was considered. Total costs at the health facility were US$ 26.65 (IQR 24.94) or 54.33 (IQR 49.09) for the whole clinical episode. Bootstrapping the estimates only slightly reduced the mean values.

Women who experienced two clinical episodes incurred costs ranging from US$ 18.91 to US$ 108.66 (Table 4). Of the 5 women who had more than one episode, two had two *P*. *falciparum* infections, two had one *P*. *vivax* episode and one had a *P*. *vivax* and a *P*. *falciparum* infection.

**Table 4:** Total costs incurred by women who experienced more than one episode during pregnancy.

Total cost (US$)	Number of episodes	Parasite types	Outpatient/inpatient
95.21	2	*P*. *falciparum*	Inpatient
108.66	2	*P*. *falciparum + P*. *vivax*	Inpatient
64.69	2	*P*. *falciparum*	Inpatient
32.57	2	*P*. *vivax*	Outpatient
18.91	2	*P*. *vivax*	Outpatient

## Discussion

Although malaria control interventions are free of charge to users in Colombia, the economic costs incurred by pregnant women in the study were substantial. Total median costs associated with inpatient treatment of a whole malaria episode (US$ 54.33) represented the 18% of the monthly minimum salary in force in the country at the time of the study (US$ 307). Transportation costs and indirect costs (cost of time) were the largest components. The highest costs of over US$ 100 were estimated for women who experienced two inpatient episodes.

This study cannot assess whether such high costs constitute a barrier to malaria diagnosis and treatment as only women who actually received a (positive) malaria diagnosis were included. However, repeated episodes requiring admission are likely to imply substantial shocks to families’ budgets.

To our knowledge, only one previous study has estimated the costs associated with *P*. *vivax* MiP [5]. In that study, conducted in Brazil, total costs were higher than the ones estimated here: bootstrapped median costs were US$ 49.27 for outpatients and 240.78 for inpatients versus 28.93 and 54.33 in this study. The main driver of this difference was the time spent to reach the health facility, which was higher in Brazil than in Colombia, and which translated into higher indirect costs, particularly for inpatients, despite the lower value of time used for Brazil than for Colombia (monthly value of 237 US$ for Brazil and of 307 US$ for Colombia). All remaining parameters estimated in the two studies were similar. Even though the two health systems are very different, malaria is considered as a disease of public health interest in both countries, and prevention and treatment are provided free of charge to patients.

All but one women in this study belonged to the first level of the subsidised system, suggesting that our sample was constituted by the poorest individuals. Although we cannot quantify their potential impact, we can speculate that costs incurred by these women are likely to represent a significant economic burden to their limited household budgets.

Importantly, in the context of this study the challenges faced by women when seeking treatment are likely to go beyond the estimated costs. Armed conflict has a detrimental impact on healthcare both from the demand and supply sides [34]. From the patient perspective, the journey from home to the health facility is likely to be dangerous, and people seeking treatment may have to deviate from the easiest path to avoid assaults.

In Colombia, the re-emergence of infectious diseases transmitted by vectors has been particularly important in areas with armed conflict [35]. In the “Colombian National malaria control and surveillance plan 2003–2006”, Tierralta, Córdoba, was defined as an area where armed conflict has exacerbated the burden of malaria due to forced displacement of the population; either a partial or total suspension of vector control activities; challenges of diagnosis and treatment; and deterioration of the environment leading to the formation of mosquitos breeding sites [36].

There has been a high proportion of victims of forced displacement in Tierralta, including the Embera Katío people who were forced to leave for several reasons: (1) the armed conflict for the control of the land for the production and distribution of illicit drugs; (2) the illegal exploitation of natural resources, mainly wood and (3) the construction of the dam Urrá I in the year 2000 [37]. There is no census reporting the exact number of displaced people; however, it is clear that the majority of displaced families lived in poverty [38]. For those who remained in the protected area, the construction of the dam resulted in serious constrains to access to food (fish in particular) and in additional limitations on movement imposed by the armed groups: these factors led to a substantial socio-economic collapse of that community [38].

In our sample, nearly 25% of women were indigenous. Malaria and the costs associated with its management are potentially only the tip of the iceberg of the problems incurred by Embera Katío community, who have suffered the violation of their rights to the land, their economic independence, education, and their culture. Currently interventions are aimed at preserving the culture and the rights of this indigenous population as planned by the Plan de Salvaguarda Etnica Pueblo Embera Katío del Alto Sinú [38].

Despite several challenges, the Colombian health system is making strong efforts to improve access to quality health care. Colombia is not far from achieving universal health care insurance: healthcare out-of-pocket expenditures have decreased consistently, and the population reports fewer unmet health needs and greater satisfaction with healthcare services following the 1993 reforms [39]. As in any context, the last mile is the most difficult to cover and the area where this study was conducted encompasses all main possible challenges: malaria endemicity, armed conflict, rural areas and minorities.

Importantly, the peace process in Colombia, which started in 2012 with negotiations between the leaders of the *guerilla* and the government and having its climax in August 2016 with the cease-fire and end of hostilities, should have a positive impact also on the health system and on access to health services in conflict areas [40].

This study has one main limitation. Some women were interviewed several months (up to a maximum of 9) after experiencing the malaria episode, which is likely to have resulted in recall bias in our estimates. Recall bias in self-reported expenditure has been described to translate into either underestimation or overestimation [41]. In this study, as questions were focused on costs incurred on a specific episode and not on recurrent expenditures, estimates are more likely to be underestimated as affected by “forgetting”. For this reason, the economic costs reported in this study should be interpreted as a lower bound of the real economic cost associated with MiP. In addition, women were interviewed only once limiting the possibility of estimating more precisely the whole cost associated with malaria during pregnancy and post-partum.

A few studies have explored the economics of malaria in Colombia. In 2006, costs of malaria treatment were estimated as part of the cost-effectiveness analysis of two alternative strategies for malaria control [7]. One strategy was constituted by the activities of the National Programme, the other by the integration of an educational strategy, the “Integrated Alternative” (IA) into the national program in Buenaventura on the Pacific coast of Colombia. Average household malaria treatment cost (including direct and indirect cost) in the area where the National Programme was implemented was US$36.2 and US$28.4 in the IA area (1998 figures).

Malaria incidence appeared to be associated with income and with occupational status in Colombia in a study that tested Becker’s human capital theory and its further developments by Popkin and Schutz [8]. An in-depth study estimated the average cost per malaria case in Santa Cruz (rio Naya, Colombia) as US$17.30, over 90% of which were indirect costs. The loss corresponded to the value of 5.6 days of work paid at the minimum monthly wage (year 1986) [6]. Finally, labour reallocation within the family was assessed to be the most significant economic consequence of malaria, with women substituting for men in the fields when men are affected by the disease [9].

Despite the valuable information existing on socio-economic aspects of malaria in Colombia, no study has examined MiP. The present study fills this gap by estimating the economic burden of MiP that can inform malaria elimination initiatives in Colombia [42].

## Supporting information

S1 Checklist STROBE checklist. (DOC)
